# Prevention of post-cardiac surgery vitamin D deficiency in children with congenital heart disease: a pilot feasibility dose evaluation randomized controlled trial

**DOI:** 10.1186/s40814-020-00700-3

**Published:** 2020-10-22

**Authors:** James Dayre McNally, Katie O’Hearn, Dean A. Fergusson, Jane Lougheed, Dermot R. Doherty, Gyaandeo Maharajh, Hope Weiler, Glenville Jones, Ali Khamessan, Stephanie Redpath, Pavel Geier, Lauralyn McIntyre, Margaret L. Lawson, Tara Girolamo, Kusum Menon, on behalf of the Canadian Critical Care Trials Group

**Affiliations:** 1 Department of Pediatrics, Division of Critical Care, University of Ottawa, Ottawa, Canada; 2 CHEO, 401 Smyth Road, Ottawa, ON K1H 8L1 Canada; 3 Children’s Hospital of Eastern Ontario Research Institute, Ottawa, Canada; 4 Department of Medicine, University of Ottawa, Ottawa, Canada; 5 Clinical Epidemiology Program, Ottawa Hospital Research Institute, Ottawa, Canada; 6 Department of Pediatrics, Division of Cardiology, University of Ottawa, Ottawa, Canada; 7 Children’s Health Ireland at Temple Street, Dublin, Ireland; 8 Department of Pediatric Surgery, Division of Cardiovascular Surgery, University of Ottawa, Ottawa, Canada; 9 School of Human Nutrition, Faculty of Agricultural and Environmental Sciences, McGill University, Montreal, Canada; 10 School of Medicine, Department of Biomedical and Molecular Sciences, Queen’s University, Kingston, Canada; 11 Euro-Pharm International Canada Inc., Montreal, Canada; 12 Department of Pediatrics, Division of Neonatology, University of Ottawa, Ottawa, Canada; 13 Department of Pediatrics, Division of Nephrology, University of Ottawa, Ottawa, Canada; 14 Department of Medicine (Division of Critical Care), Ottawa Hospital Research Institute (OHRI), University of Ottawa, Ottawa, Canada; 15 Department of Pediatrics, Division of Endocrinology, University of Ottawa, Ottawa, Canada

**Keywords:** Vitamin D deficiency, Congenital heart disease, Critical care, Pediatric intensive care unit, Cholecalciferol, High-dose, Dose evaluation trial

## Abstract

**Background:**

The vast majority of children undergoing cardiac surgery have low vitamin D levels post-operative, which may contribute to greater illness severity and worse clinical outcomes. Prior to the initiation of a large phase III clinical trial focused on clinical outcomes, studies are required to evaluate the feasibility of the study protocol, including whether the proposed dosing regimen can safely prevent post-operative vitamin D deficiency in this high-risk population.

**Methods:**

We conducted a two-arm, double-blind dose evaluation randomized controlled trial in children requiring cardiopulmonary bypass for congenital heart disease. Pre-operatively, participants were randomized to receive cholecalciferol representing usual care (< 1 year = 400 IU/day, > 1 year = 600 IU/day) or a higher dose approximating the Institute of Medicine tolerable upper intake level (< 1 year = 1600 IU/day, > 1 year = 2400 IU/day). The feasibility outcomes were post-operative vitamin D status (primary), vitamin D-related adverse events, accrual rate, study withdrawal rate, blinding, and protocol non-adherence.

**Results:**

Forty-six children were randomized, and five withdrew prior to surgery, leaving 41 children (21 high dose, 20 usual care) in the final analysis. The high dose group had higher 25-hydroxyvitamin D concentrations both intraoperatively (mean difference + 25.9 nmol/L; 95% CI 8.3–43.5) and post-operatively (mean difference + 17.2 nmol/L; 95% CI 5.5–29.0). Fewer participants receiving high-dose supplementation had post-operative serum 25-hydroxyvitamin D concentrations under 50 nmol/L, compared with usual care (RR 0.31, 95% CI 0.11–0.87). Post-operative vitamin D status was associated with the treatment arm and the number of doses received. There were no cases of hypercalcemia, and no significant adverse events related to vitamin D. While only 75% of the target sample size was recruited (limited funding), the consent rate (83%), accrual rate (1.5 per site month), number of withdrawals (11%), and ability to maintain blinding support feasibility of a larger trial.

**Conclusions:**

Pre-operative daily high-dose supplementation improved vitamin D status pre-operatively and at time of pediatric ICU admission. The protocol for a more definitive trial should limit enrollment of children with at least 30 days between randomization and surgery to allow adequate duration of supplementation or consider a loading dose.

**Trial registration:**

ClinicalTrials.gov, NCT01838447. Registered on April 24, 2013

## Background

Congenital heart disease (CHD) is a common condition occurring in 1 per 100 births. Many of these lesions are pathological, necessitating cardiac surgery to prevent death and improve short- and long-term quality of life [1]. Due to underlying cardiac lesions and the deleterious effects of complex operative procedures, these children are at risk for post-operative mortality and morbidity secondary to pronounced systemic inflammatory responses, respiratory failure, myocardial dysfunction, acute kidney injury, and infection [2–5]. Interest remains in the identification of interventions that reduce or lessen perioperative morbidity and in doing so improve quality of life and functional status and save health care spending.

Recently, a significant body of work has emerged suggesting vitamin D to be a potentially modifiable risk factor in adult and pediatric critical care settings [6–8]. A systematic review of 17 observational studies reporting on 25-hydroxyvitamin D (25OHD) concentrations in the general pediatric intensive care unit (ICU) setting concluded that 55% of critically ill children worldwide have admission 25OHD concentrations below the accepted target threshold of 50 nmol/L required for optimal axis functioning [9]. Consistent with its pleiotropic actions, these studies also demonstrated poor vitamin D status to be associated with dysfunction of multiple organs central to recovery from critical illness (cardiac, respiratory, immune) and reduced survival [10–13]. Supplementing this work, four studies focused on children with CHD report that between 50 and 90% have post-operative serum 25OHD concentrations below 50 nmol/L [14–17]. Further, three of the studies reported statistically significant associations between serum 25OHD concentrations and post-operative cardiovascular dysfunction [14, 15, 17]. Low post-operative 25OHD concentrations appear to be secondary to a combination of low to borderline normal pre-operative 25OHD concentrations and a documented ~ 40% intraoperative decline as a direct result of cardiopulmonary bypass [15, 17–20]. Observational work by multiple groups describes an abrupt 40% decline in 25OHD concentrations during pediatric CHD surgery, which coincided with the initiation of cardiopulmonary bypass [15, 17]. Similarly, a 35% decline in 25OHD concentrations has been observed in adults following the initiation of cardiopulmonary bypass [21]. The available literature suggests that usual low-dose daily supplementation based on the recommended daily allowance issued by the Insititute of Medicine is inadequate to maintain post-operative 25OHD concentrations above 50 nmol/L in the CHD population [19].

Vitamin D is an inexpensive and readily available intervention, and there has been considerable interest in evaluating whether the application of alternative supplementation approaches could lead to improved outcomes in the ICU or cardiac surgical populations. Meta-analysis of pilot adult randomized controlled trials (RCTs) providing high-dose supplementation following ICU presentation suggests clinical benefit [22, 23], coinciding with the initiation of two large multicenter adult RCTs both evaluating enteral loading doses of 540,000 IU (NCT03096314, NCT03188796) [24, 25]. Inspection of the pediatric ICU and cardiac surgical literature identifies only two pilot studies of vitamin D supplementation, with one on children with burns [26, 27]. The second more relevant study by Sahu and colleagues administered a single 10,000 IU/kg vitamin D loading dose prior to cardiac surgery in children with Tetrology of Fallot, demonstrating significant improvements in post-operative vitamin D status compared to control (61 vs. 30 nmol/L) [27]. While single high-dose supplementation, pre- or post-operatively, is a valid option, the CHD patient is unique among the pediatric ICU population in that the majority are admitted following a surgery planned days, weeks, or months prior. Consequently, it may be possible to prevent post-operative vitamin D deficiency using an alternative pre-operative daily high-dose strategy.

In addition to the usual care based on Recommended Daily Allowance, the Institute of Medicine also provided a second higher dose intended to generate significantly higher vitamin D status, while avoiding toxicity, referred to as the tolerable upper intake level (1000–4000 IU/day, age-specific) [19]. While the 25OHD concentrations achieved and the safety of the alternative high-dose regimen has been thoroughly evaluated in healthy children, performance in other populations including children with CHD remains unknown [28, 29]. Prior to a large phase III trial evaluating for improved clinical outcomes, the feasibility of a study protocol based on the pre-operative administration of the daily tolerable upper intake level should be evaluated in a pilot study.

This manuscript presents the findings of a pilot dose evaluation randomized controlled trial focused on feasibility. The primary feasibility objective of this trial was to determine whether a pre-operative regimen of daily high-dose (HD) vitamin D3 (cholecalciferol) based on the Institute of Medicine age-specific tolerable upper intake level significantly reduces post-operative vitamin D deficiency following CHD surgery with cardiopulmonary bypass, when compared with usual care (UC). Additional protocol-specific feasibility objectives evaluated whether HD supplementation results in additional clinical and biochemical vitamin D-related adverse events (hypercalcemia, hypercalciuria, nephrocalcinosis) and generalizability feasibility outcomes related to accrual, blinding, study withdrawal, and protocol non-adherence.

## Methods

### Study design

This trial is reported according to the Consolidated Standards of Reporting Trials (CONSORT, Additional file 1) extension for randomized pilot and feasibility trials [30, 31]. We conducted a single-center, double-blind, phase II dose evaluation pilot RCT from July 2013 to December 2015 at a Canadian tertiary center. The study rationale, design, and protocol have been previously published [32]. Ethical approval was obtained from the Children’s Hospital of Eastern Ontario Research Ethics Board (#13/03E), and regulatory approval was obtained from Health Canada (CTA #161404). Written informed consent was obtained from all participants before the initiation of study procedures, with written assent obtained where applicable. The study protocol was registered on ClinicalTrials.gov by J.D. McNally on April 24, 2013 (NCT01838447).

#### Participants

Patients aged 36 weeks corrected gestational age to 17 years with CHD who required surgical correction with cardiopulmonary bypass within the next 12 months were included. Exclusion criteria were cardiac or gastrointestinal disease-preventing enteral feeds or drug administration prior to surgery, confirmed or suspected William’s syndrome, proposed surgery to take place at another hospital, surgical correction without the need for cardiopulmonary bypass, and born at less than 32 weeks gestational age.

#### Randomization

Children with CHD meeting the eligibility criteria were recruited pre-operatively following diagnosis and confirmation of the need for surgery from ambulatory clinics (cardiology and cardiovascular surgery), general inpatient wards, and both neonatal and pediatric ICUs. Patients were randomized by the pharmacy using 1:1 in permuted blocks of four and stratified by age (< 1 or > 1 year) and expected time to surgery (< 2 months or > 2 months). Patients < 1 year of age were also stratified by feeding status (breast- or formula-fed). Randomization lists were generated by the Ottawa Hospital Method Centre and were only available to site research pharmacists. Blinding was maintained using indistinguishable interventions (identical vials, volume, color, taste, consistency, and smell). With the exception of the research pharmacists, all study personnel, clinical staff, patients, and families were blinded to the group assignment.

#### Intervention

The study drug was provided in different dosages of vitamin D3 (cholecalciferol) and placebo by Euro-Pharm International Canada Inc. Patients received a daily enteral dose of the study drug from the time of study enrollment to the day of CHD surgery. The HD and UC dosing regimens were both modeled on age-specific intake levels recommended by the Institute of Medicine (Table 1) [19]. Patients in the high-dose arm received an enteral daily dose based on the Institute of Medicine tolerable upper intake level, equivalent to 1600 IU (< 1 year) or 2400 IU (1–17 years) per day. The tolerable upper intake level is intended to significantly elevate serum 25OHD concentrations above the 50 nmol/L threshold established for bone health, while minimizing the risk of vitamin D toxicity (hypercalciuria, hypercalcemia). Given the expected 40% intraoperative decline in 25OHD concentrations due to the use of cardiopulmonary bypass, pre-operative 25OHD concentrations above 90 nmol/L are required to maintain post-operative 25OHD concentrations above 50 nmol/L. The HD dosing regimen was selected to achieve a pre-operative 25OHD target of 90 nmol/L [32]. Patients randomized to the usual care arm received the Adequate Intake doses for infants or recommended daily allowance for children > 1 year of age. The Adequate Intake and Recommended Dietary Allowance  daily doses were chosen with the goal of gradually (over approximately 2 months) achieving blood 25OHD concentrations at or slightly above the 50 nmol/L threshold associated with good bone health. With the exception of study intervention, the anesthetic, surgical, and ICU management was left to the discretion of the anesthesiologists, perfusionists, cardiovascular surgeons, and intensivists.

**Table 1:** Vitamin D dosing regimen

Age group	Volume, mL	Usual care group		High dose group	
		IU/day	Vial concentration, IU/mL	IU/day	Vial concentration, IU/mL
*Breastfed infant children > 12 months of age*					
0–1 year^b^	1	400	400	1600	1600
1–17 years	1	600	600	2400	2400
*Formula-fed infant (< 12 months of age)*					
0–1 year^b^	1	None^a^	Placebo (0)	1200	1200

^**a**^Does not include the vitamin D intake from formula (400 IU/day)

^**b**^Vitamin D dosing was not increased for children who reached 1 year of age after initiating study drug

### Study feasibility outcome measures

The primary feasibility outcome was the proportion of participants with an immediate post-operative serum 25OHD concentration under 50 nmol/L (equivalent to 20 ng/mL), determined using a validated LC-MS/MS assay, as previously described [32, 33]. Immediate post-operative serum 25OHD concentration was determined after the final separation from cardiopulmonary bypass and collected at the time of pediatric ICU admission bloodwork. As cardiac surgery and cardiopulmonary bypass are known to significantly reduce blood 25OHD levels, we also report on vitamin D status intraoperatively, just prior to the initiation of bypass. Additional feasibility outcome measures specific to the proposed study dosing regimens included the three well-studied vitamin D-related adverse events: hypercalciuria, hypercalcemia, and nephrocalcinosis. Additional non-specific outcome measures used to evaluate RCT feasibility more broadly included accrual rate, study withdrawal rate, non-adherence to the study protocol, and ability to maintain allocation and blinding.

### Exploratory clinical outcomes

Common clinical outcomes were collected and compared between the groups, as described in the protocol paper. Post-operative clinical outcomes included mortality, fluid intake, hypocalcemia, catecholamine infusion requirements, arrhythmias, positive bacteria culture, renal failure, and duration of mechanical ventilation, pediatric ICU, and hospital stay. The Pediatric Risk of Mortality (PRISM) score was also calculated, as previously reported, with the exception that children with cyanotic lesions (e.g., single ventricle) were not penalized for low PaO_2_ [34].

### Research sample collection

For the primary study objective, blood was collected immediately post-operatively (after the final separation from cardiopulmonary bypass, with pediatric ICU admission bloodwork), processed and stored at − 80 °C for the analysis of 25OHD at study close-out. To address the secondary and exploratory objectives, additional blood was collected intraoperatively (just prior to the initiation of cardiopulmonary bypass) and on post-operative days (POD) 1, 3, 5, and 10 (or at the time of pediatric ICU discharge if the patient was discharged before day 10) (see schema of research sample collection timing, Additional file 2). Blood samples were collected through arterial or venous catheters or at the time of clinically indicated bloodwork. As some patients often do not have arterial or venous catheters in place after day 2, the research samples were not collected unless venipuncture was planned for clinically indicated bloodwork. In addition to blood, urine samples were collected in the operating room and on POD1 to measure calcium and creatinine ratios and monitor for hypercalciuria.

### Safety monitoring

Real-time safety monitoring of participants’ blood 25OHD and calcium concentrations  was performed at the request of the Research Ethics Board and Data Safety Monitoring Board. First, for participants started on study drug before their scheduled pre-surgical appointment, 25OHD and ionized calcium measurement were added to the bloodwork performed at their scheduled pre-surgical appointment (2 to 3 weeks prior to surgery). Second, participants who were on study drug for more than 6 months also had mid-treatment blood collected to monitor 25OHD and ionized calcium. The 25OHD and ionized calcium results from the mid-treatment and pre-surgical bloodwork were reviewed by the study safety officer in a blinded manner to evaluate for signs of vitamin D toxicity. Hypercalcemia was defined as an ionized calcium level above 1.40 mmol/L or 1.45 mmol/L for patients < 2 months of age [35–37]. In the absence of hypercalcemia, 25OHD concentrations above 200 nmol/L and 250 nmol/L called for either a 50% reduction or discontinuation of the study drug, respectively. Third, intraoperative and POD1 urine calcium to creatinine ratios were measured in real time by the clinical laboratory and monitored by a study safety officer. Age-based thresholds, as previously published [32], were used to evaluate for hypercalciuria (Additional file 3).

### Compliance

Adherence to the intervention was evaluated by requesting that caregivers (i) document daily administration of the study drug in a calendar (print) and (ii) return unused study drug to the research team on the day of surgery. Compliance was determined based on the volume of drug provided vs. what was returned and/or the number of doses recorded in the compliance calendar. For inpatients, compliance was determined based on the number of administered doses documented on the patient’s medication record in their hospital chart. Compliance data was considered in the evaluation of the relationship between the duration of study treatment and post-operative 25OHD concentrations.

### Sample size

The study sample size was calculated using the feasibility objective focused on the prevention of post-operative vitamin D deficiency with the proposed HD supplementation strategy. Based on the findings from observational studies and clinical trials on healthy children receiving adequate intake or recommended daily allowance, it was estimated that ≤ 40% of participants in the UC arm would have post-operative 25OHD concentrations  above 50 nmol/L. Similarly, through an evaluation of pediatric studies administering cholecalciferol doses approximating the Institute of Medicine upper tolerable intake level, it was estimated that ≥ 80% of participants in the HD arm would achieve post-operative levels above 50 nmol/L. To detect an absolute difference between arms of ≥ 0.4 with *α* = 0.05 and *β* = 0.8, the study sample size would need to be 28 per arm. After adjustment for up to a 10% dropout, the final sample size was set at 62 (total).

### Evaluation of protocol feasibility

The criteria were established to assist with the determination of the feasibility of a phase III trial utilizing the existing protocol, or whether adjustments were necessary. For the vitamin D status outcome, a 40% absolute decrease in the frequency of post-operative vitamin D deficiency was set, consistent with the sample size calculation. In addition, the investigators recognized an ideal state where ≥ 75% of the HD arm achieved immediate post-operative 25OHD above 50 nmol/L, with subgroup analysis intended to identify subgroups where a different dosing regimen might be required. For vitamin D-related adverse events, the criteria for adjustment of the HD regimen were set as the occurrence of persistent hypercalcemia and/or nephrocalcinosis in the HD group, with 25OHD concentrations above 200 nmol/L. Protocol adherence was considered acceptable if the major protocol deviations related to study drug administration or safety procedures occurred in less than 20% of enrolled patients. The acceptable level set for both study dropout and unblinding rates was set at ≤ 10%. Finally, as sufficient funding was available for only a 30-month recruitment period, the target accrual rate for the study was set as 2 to 2.5 patients per month (per site). While ideal, the target accrual rate above 2 patients per month exceeds that observed in many phase III multicenter pediatric ICU studies; therefore, the acceptable accrual rate to establish feasibility was set at 1.5 patients per month.

### Statistical analysis

Baseline demographic data are presented as proportions for dichotomous variables while continuous variables are presented as either means with standard deviations for normally distributed variables or medians with interquartile range (IQR). Differences in the primary outcome measure (proportion with 25OHD under 50 nmol/L) between the treatment groups were evaluated using relative risk, reported with 95% confidence intervals (CI) in an intention-to-treat analysis. Secondary analyses were evaluated and reported based on data type. Continuous outcome measures were evaluated using the *t* test or the Wilcoxon sign rank test, with differences between the groups reported as the mean difference with 95% CI. Dichotomous secondary outcome measures (e.g., hypercalcemia, hypercalciuria) were compared using Fisher’s exact, chi-square, or relative risk (RR) with 95% CI. Outcome measures that represent time to event (e.g., time to extubation, pediatric ICU, and hospital length of stay) were analyzed using the log rank test. Spearman correlation coefficients and linear regression models were used to evaluate how (i) duration of therapy and (ii) the number of study doses received (incorporating compliance) influenced the relationship between vitamin D status and treatment arm. Analyses were performed using the SAS software (Copyright SAS Institute Inc., Cary, NC, USA), and a *p* value less than 0.05 will be considered statistically significant.

## Results

**Fig. 1:**
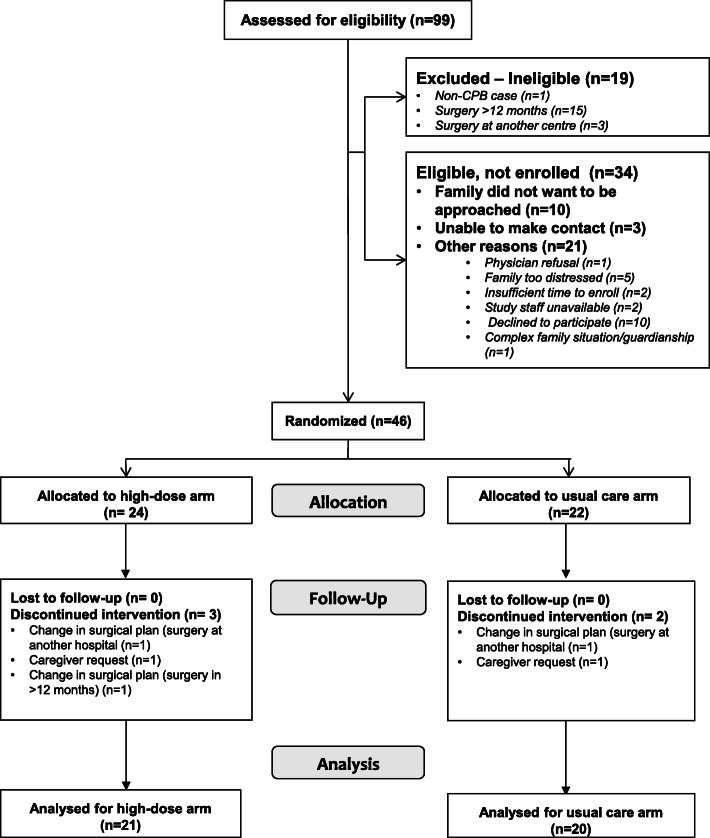
CONSORT diagram. CPB, cardiopulmonary bypass

Ninety-nine children referred to the cardiovascular surgery team from July 2013 to December 2015 were screened for eligibility, with 46 randomized (CONSORT diagram, Fig. 1). Five patients were withdrawn pre-operatively, leaving 41 patients in the final analysis. In three cases, the research team initiated study withdrawal as the surgical management plan was altered and the patient no longer met the study eligibility: significant delay in the timing of surgery (*n* = 1) and surgery would occur at another center (*n* = 2). The two remaining patients were withdrawn at the request of the caregiver. No patients were withdrawn due to side effects of the study drug. Baseline patient characteristics are shown in Table 2, with no clinically important differences evident between the groups. Collection of pediatric ICU admission blood samples for immediate post-operative 25OHD measurements was possible for all 21 patients in the HD arm and 19 study participants in the UC arm.

**Table 2:** Comparison of demographics by intervention arm

Patient characteristic	High dose (*n* = 21)	Usual care (*n* = 20)
Age (months), median (IQR)	4.6 (1.0–45.6)	3.8 (0.8–35.6)
Weight (kg), median (IQR)	5.4 (4.2–13.4)	5.8 (3.6–12.9)
Male sex, *n* (%)	10 (47.6%)	13 (65.0%)
Enrolment location, *n* (%)		
Cardiology	15 (71.4%)	15 (75.0%)
NICU	4 (19.0%)	5 (25.0%)
PICU	2 (9.5%)	0 (0.0%)
Surgery season, *n* (%)		
Winter	6 (28.6%)	5 (25.0%)
Spring	3 (14.3%)	2 (10.0%)
Summer	6 (28.6%)	8 (40.0%)
Fall	6 (28.6%)	5 (25.0%)
RACHS, median (IQR)	2 (2–3)	3 (2–4)
Ethnicity, *n* (%)		
Caucasian	14 (66%)	14 (70.0%)
Aboriginal	1 (5%)	1 (5.0%)
Asian	0 (0%)	3 (15.0%)
African/West Indian	2 (10%)	1 (5.0%)
Unknown or others	4 (19%)	1 (5.0%)
Other active medical conditions, *n* (%)	7 (33.3%)	7 (35.0%)
*Genetic*	*5 (71.4%)*	*3 (42.9%)*
*Chronic lung*	*1 (14.3%)*	*2 (28.6%)*
*Endocrine*	*1 (14.3%)*	*1 (14.3%)*
*Others*	*3 (42.9%)*	*4 (57.1%)*
Expected time to surgery at enrolment, *n* (%)		
< 2 months	18 (86%)	18 (90%)
> 2 months	3 (14%)	2 (10%)
Children ≤ 1 year, *n* (%)		
Breastfed	10 (83%)	9 (75%)
Formula-fed	2 (17%)	3 (25%)

### Feasibility objectives: vitamin D outcomes

**Fig. 2:**
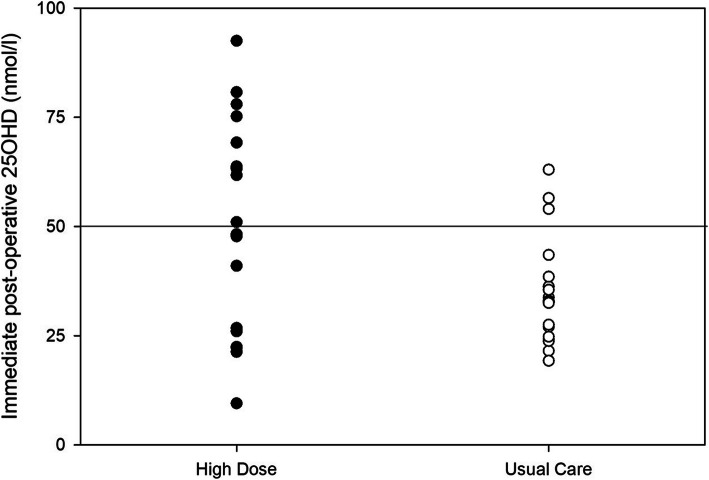
Box plot of post-operative 25OHD concentrations in the high-dose and usual care arm

For the primary outcome, the proportion of participants with a serum 25OHD under 50 nmol/L immediately post-operatively, the HD arm was significantly lower (43% vs. 84%) compared to UC. This decline in proportion represents a calculated RR of 0.31 (95% CI 0.10–0.87). The UC study participant without a pediatric ICU admission blood sample had a 25OHD concentration of 21 nmol/L on POD1. When 25OHD was evaluated continuously, the HD group had significantly higher immediate post-operative serum concentrations (52.0 ± 23.3 vs. 34.8 ± 12.0 nmol/L) compared to UC, with a calculated mean difference of 17.2 nmol/L (95% CI 5.5–29.0) (Fig. 2). Table 3 provides the serum 25OHD concentrations by group for the remaining post-operative time points and the proportion of participants in each group who were vitamin D deficient. The 25OHD concentrations in the HD group remained consistently higher throughout the study period, with the difference between the groups achieving significance on POD1 and POD3.

**Table 3:** Intraoperative and immediate post-operative 25OHD concentrations

Time point (*n* = high dose, low dose)	25OHD (nmol/L)		*N* (%) VDD	
	High dose	Usual care	High dose	Usual care
Intraoperative—prior to the initiation of CPB (*n* = 21, 20)	81.5 (36.5)^a^	55.5 (13.8)	5 (24%)	7 (35%)
Immediately post-operative at PICU admission (*n* = 21, 19)	52.0 (23.3)^a^	34.8 (12.0)	9 (43%)	16 (84%)
POD1 (*n* = 20, 19)	53.8 (23.5)^a^	33.0 (17.8)	9 (45%)	15 (79%)
POD3 (*n* = 9, 16)	55.0 (28.3)^a^	34.3 (11.5)	4 (44%)	14 (88%)
POD5 (*n* = 8, 12)	58.3 (38.3)	35.8 (13.5)	4 (50%)	9 (75%)

Data are mean (SD)

*CPB* Cardiopulmonary bypass, *VDD* Vitamin D deficiency (25OHD < 50 nmol/L), *PICU* Pediatric intensive care unit, *POD* Post-operative day

^**a**^Significantly higher in HD than UC; POD10/discharge 25OHD concentration only obtained for 6 patients, data not shown

**Fig. 3:**
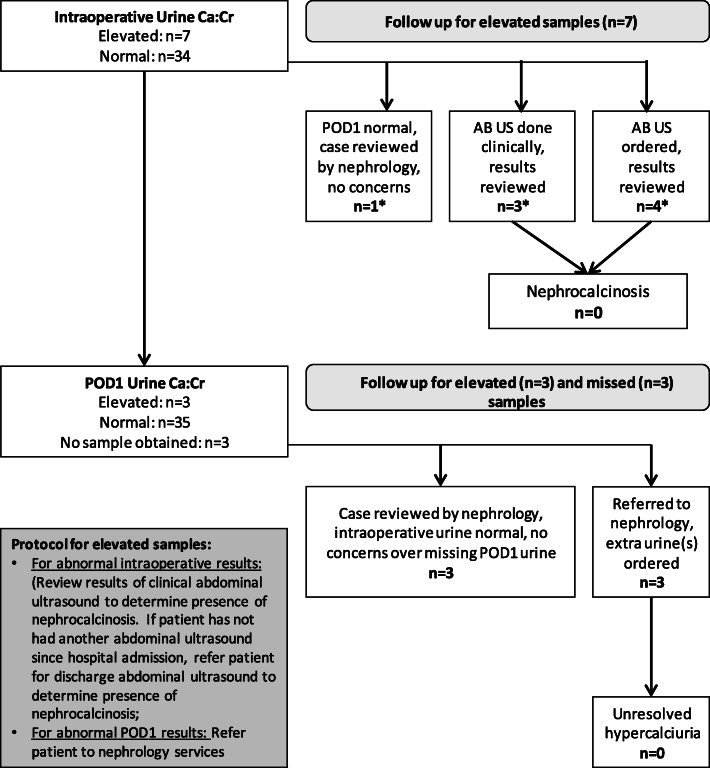
Evaluation of hypercalciuria. *Numbers add up to more than 7 because one patient had both a clinical and discharge ultrasound. AB US, abdominal ultrasound; ca:cr, calcium to creatinine ratio; POD1, post-operative day 1

There were no cases of hypercalcemia documented on any of the mid-treatment or pre-surgical measurements. Similarly, no cases of hypercalcemia were observed in the operating room immediately before surgery. Post-operatively, two patients exhibited transient hypercalcemia (< 24 h) and both were in the UC group. The comparison of mean ionized calcium concentrations by study group assignment over the post-operative course did not find any suggestion of inappropriate elevation of ionized calcium in the HD group (Table 4). As shown in Table 4, transient hypercalciuria was documented in some study participants at each time point, equally between the study groups. First, 7 study participants (17%) had elevated intraoperative urine calcium to creatinine ratios (UC = 3, HD = 4). Six of these participants had abdominal ultrasounds confirming the absence of nephrocalcinosis, with the seventh (HD group) having a resolution of hypercalciuria by POD1; due to the short duration of study drug administration (3 days), nephrology advised that no ultrasound was necessary. All but three study participants had urine calcium concentrations measured on POD1, with three cases of hypercalciuria identified (UC = 2, HD = 1). Patients with hypercalciuria were evaluated by nephrology, and treatment follow-up was tailored to the clinical scenario; in all cases, hypercalciuria resolved (often with discontinuation of furosemide). The three patients for whom a POD1 urine sample was not obtained were reviewed by nephrology. In all cases, the intraoperative urine calcium to creatinine ratio was normal, and the study nephrologist did not require additional follow-up (Fig. 3). There were no clinically relevant adverse events assessed as being related to vitamin D during the conduct of the trial.

**Table 4:** Pre- and post-operative blood and urine calcium measurements

	High dose (*n* = 21)	Usual care (*n* = 20)	*p* value
Hypercalciuria			
Enrolment urine calcium to creatinine ratio above age-based threshold, *n* (%) (*n* = 6, 6)	0	1	1.0
Intraoperative urine calcium to creatinine ratio above age-based threshold, *n* (%) (*n* = 21, 20)	4 (19%)	3 (15%)	0.73
Post-operative day 1 urine calcium to creatinine ratio above age-based threshold, *n* (%) (*n* = 18, 20)	1 (4.8%)	2 (10.0%)	0.61
Hypercalcemia			
Mid-treatment ionized calcium concentration (mmol/L) (*n* = 3, 0)^a^	1.17 ± 0.08	n/a	n/a
Pre-surgical ionized calcium concentration (mmol/L) (*n* = 12, 14)	1.18 ± 0.04	1.20 ± 0.09	0.42
Intraoperative ionized calcium concentration (mmol/L) (*n* = 21, 20)	1.14 ± 0.07	1.14 ± 0.08	0.912
Post-operative ionized calcium			
PICU admission (*n* = 20, 20)	1.19 ± 0.09	1.20 ± 0.08	0.66
POD1 (*n* = 21, 20)	1.20 ± 0.07	1.18 ± 0.10	0.28
POD3 (*n* = 8, 13)	1.19 ± 0.07	1.26 ± 0.08	0.04
POD5 (*n* = 7, 10)	1.18 ± 0.05	1.18 ± 0.07	0.98
Transient (< 24 h) hypercalcemia during PICU admission, *n* (%)	0 (0%)	2 (10.0%)	0.23

Hypercalcemia was defined as an ionized calcium level > 1.40 mmol/L or > 1.45 mmol/L for patients < 2 months of age. Hypercalciuria was defined previously published age-based thresholds [28]

*PICU* Pediatric intensive care unit, *POD* Post-operative day

^a^Mid-treatment ionized calcium was only checked for children with an expected study drug therapy duration of > 2 months

### Feasibility outcomes: accrual, withdrawal, and protocol adherence

A total of 46 patients were randomized over a period of 30 months for an average accrual rate of 1.5 patients per month. Of the 80 patients who met eligibility, 56 (70%) were approached by research staff and 46 agreed to participate, for a consent rate of 82%. The reasons families were not approached are outlined in Fig. 1. The most common reasons patients were not recruited include family not wishing to be approached about research (*n* = 10), family declining to participate after discussion (*n* = 10), and health care provider assessment that the family was too distressed (*n* = 5). Only 74% of the target sample size was recruited, as the study was scheduled to terminate after 2.5 years due to available funding.

Of the 46 patients randomized, 5 were withdrawn (11%). Of these, 2 (4%) were at the request of the caregivers, with the remaining 3 (6.5%) withdrawn by the study staff due to a change in surgical plan from single ventricle at our center to biventricular repair at another site. There were 22 minor protocol violations over the course of the study, including patients not returning unused study drug at the time of surgery (*n* = 4), compliance calendar not returned (*n* = 6), minor medication dispensing error (*n* = 3), study procedures omitted or conducted out of sequence (*n* = 6) and others (*n* = 3). The minor medication error and study procedures had no effect on patient safety, patient rights, or integrity of study data. Details on the protocol violations are provided in Additional file 4. There were no incidents of accidental unblinding, with blinding broken intentionally for the one patient with an elevated mid-treatment 25OHD level (254 nmol/L).

### Exploratory outcomes: 25OHD concentrations at other study time points

Evaluation of available mid-treatment and pre-surgical bloodwork by the safety officer only identified one patient with a serum 25OHD concentration above the 200 nmol/L threshold (254 nmol/L). A review of drug history with the caregiver identified that the patient was also receiving a previously undisclosed “immune tonic” containing vitamin D at the recommendation of a naturopath. The patient was determined to be in the HD group and subsequently had their study dose reduced by 50% leading to a decline in 25OHD concentration to 110 nmol/L at the time pre-surgical bloodwork 7 weeks later. The patient did not experience any adverse events potentially related to vitamin D throughout the study.

Intraoperative blood (collected just before the initiation of cardiopulmonary bypass) was available for all 41 study participants, with 25OHD concentrations higher in the HD group, when compared with UC (81.5 ± 36.5 vs. 55.5 ± 13.8 nmol/L) with a calculated mean difference of + 25.9 nmol/L (95% CI + 8.3 to + 43.5 nmol/L). The proportion of participants with vitamin D deficiency at the intraoperative time point was similar between the groups (24% in HD vs. 35% in UC), with a calculated RR of 0.77 (95% CI 0.41 to 1.43). The mean percentage intraoperative decline in serum 25OHD concentration was 36% (SD 22%), with no significant mean difference (4.5%, 95% CI 18.4 to 9.4%). We did not report on pre-enrollment 25OHD or evaluate the change in 25OHD as baseline samples were only available for 25 % (*n* = 11) of the study participants; this was due to the lack of clinically indicated bloodwork at the time of study enrollment.

### Exploratory outcomes: treatment duration, compliance, and vitamin D status

The median duration between randomization and surgery was 28 days (IQR 6, 56), with 10 (24%) having a study duration of ≥ 60 days. The number of participants enrolled for ≥ 60 days was similar between the intervention groups (HD, 4; UD, 6). Study drug and/or calendars were available to evaluate compliance in 38 study participants (93%). The median compliance rate for the full study cohort was 94% (IQR 77, 100). While the point estimate for the compliance rate was lower in the HD group, the calculated mean difference was not significant (− 8.6%, 95% CI − 21.7 to 4.5). Further, the number of study doses received in each study arm was not statistically different, with a calculated mean difference of − 2 doses (95% CI − 26 to 24 doses). After adjusting for compliance, only 41% (*n* = 17) and 17% (*n* = 7) of the participants received more than 30 and 60 doses of study drug, respectively.

A multivariate logistic regression model was used to evaluate whether there was a relationship between the primary outcome and both group assignment and duration of therapy. In the first model, while the HD arm was associated with increased odds of 25OHD > 50 nmol/L (OR 16.2, *p* = 0.01), the time duration between randomization and surgery was not statistically significant (OR 1.02 per day, *p* = 0.06). In the second model, adjusting for compliance, HD group assignment (OR 16.8, *p* = 0.02) and the increasing number of study doses received (OR 1.05 per day, *p* = 0.02) were both independently statistically associated.

**Fig. 4:**
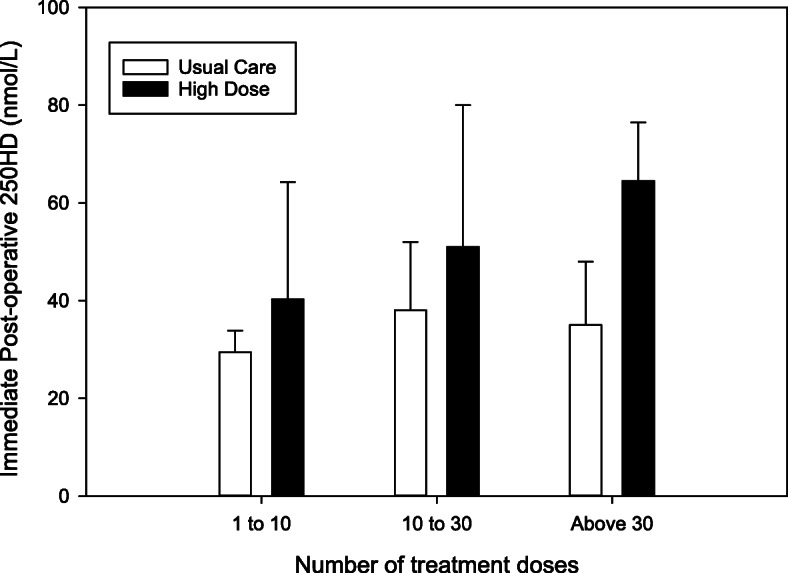
Immediate post-operative 25OHD concentrations by cumulative study drug doses

The relationship between group assignment, number of study doses received, and vitamin D status was also considering using multivariable linear regression modeling. This evaluation again confirmed that group assignment (HD + 16.2 nmol/L, 95% CI 4.3–28.22) and number of study drug doses (0.20 nmol/L per dose, 95% CI 0.04–0.36) were associated with higher post-operative 25OHD concentrations. The relationship between post-operative 25OHD concentrations, treatment arm, and number of study doses is shown graphically in Fig. 4. Only the HD participants who received more than 30 doses achieved average post-operative 25OHD concentrations above 50 nmol/L (66.8 nmol/L, SD 11.3) and were statistically different from their corresponding UC subgroup (+ 31.9 nmol/L, 95% CI 17.6–46.2).

### Exploratory outcomes: illness severity and clinical course

**Fig. 5:**
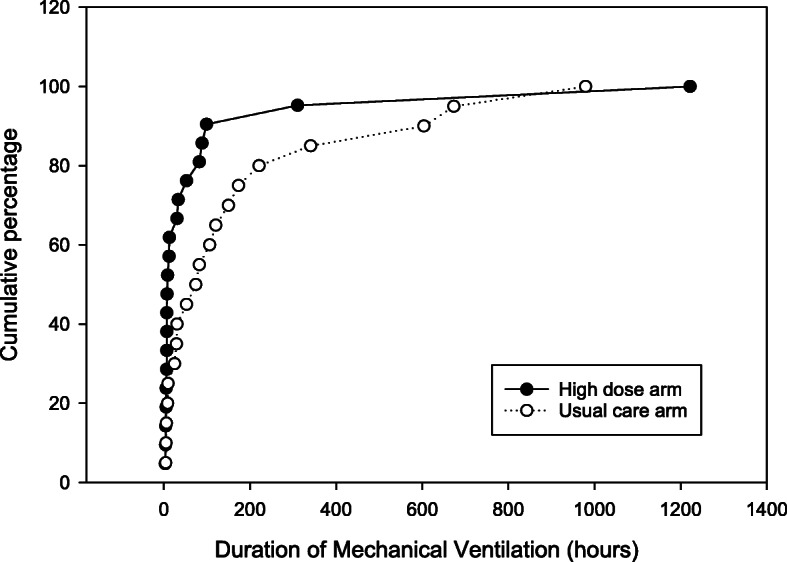
Total duration of mechanical ventilation by treatment arm

There were no deaths among study participants. Table 5 provides the results of exploratory analyses comparing pediatric ICU illness severity scores and clinical outcomes, by treatment arm. The point estimate for all measures favored the HD group, with the duration of mechanical ventilation (Fig. 5) and frequency of acute renal failure achieving statistical significance (*p* < 0.05).

**Table 5:** Clinically important outcomes

End point	High dose (*n* = 21)	Usual care (*n* = 20)	*p*
PRISM score, median (IQR)^a^	5.0 (4.0, 7.0)	7.0 (5.0, 8.5)	0.1
Total fluid intake POD1 to 3 (mL/kg), median (IQR)^b^	258 (155,382)	319 (250, 766)	0.10
Hypocalcemia, *n* (%)	8 (38.1)%	10 (50.0%)	0.44
Received catecholamines, *n* (%)	11 (52.4%)	15 (75.0%)	0.13
Post-operative arrhythmia, *n* (%)	5 (23.8%)	10 (50.0%)	0.08
Positive post-operative culture, *n* (%)	5 (23.8%)	6 (30.0%)	0.66
ARF requiring dialysis, *n* (%)	2 (9.5%)	7 (35.0%)	0.049
Length of initial MV (h), median (IQR)	7 (6, 46)	41 (9,138)	0.06
Length of MV total (h), median (IQR)	9 (6,52)	77 (17,193)	0.03
PICU length of stay, median (IQR)	3 (1,7)	7 (3.5, 13)	0.10
Hospital length of stay, median (IQR)	8 (4,13)	13.5 (7, 20)	0.11
In-hospital mortality, *n* (%)	0 (0%)	0 (0%)	–

*PRISM* Pediatric Risk of Mortality, *POD* Post-operative day, *ARF* Acute renal failure, *MV* Mechanical ventilation, *PICU* Pediatric intensive care unit

^**a**^For patients with cyanotic heart disease, PaO_2_ was not included in the PRISM score calculation as it is expected that PaO_2_ would be low for these patients

^**b**^Total fluid intake represents the fluid intake over the first 3 post-operative days

## Discussion

In recent years, a large body of observational data has emerged demonstrating that vitamin D deficiency is not only common in the pediatric ICU setting but also associated with a more protracted clinical course and increased chance of death. Further, available studies suggest that the post-operative CHD patient may be at even greater risk of vitamin D deficiency [14–17]. Unlike most critically ill patients, the post-operative CHD population is unique in that the majority are admitted following a surgery planned days, weeks, or months following presentation and diagnosis. Consequently, we hypothesized and proved in this pilot feasibility trial that pre-operative daily high-dose cholecalciferol supplementation, based on Institute of Medicine recommendations, is able to significantly reduce the post-operative vitamin D deficiency rate, when compared to usual care [32]. This trial represents one of the first vitamin D dosing studies in children with CHD [38], providing valuable information to parents and health care professionals on how to safely optimize post-operative vitamin D status, and represents the penultimate step to performing a large phase III clinical trial primarily focused on clinical outcome.

In addition to evaluating change in 25OHD concentrations post-operatively, this study also considered vitamin D-related adverse events in the assessment of feasibility. This was important as the safety data rationalizing the tolerable upper intake level originated from studies on healthy children, and children with CHD may respond differently due to critical illness, genetic abnormalities, and medications [39, 40]. First, we did not document any instances of persistent hypercalcemia post-operatively, a finding that is both reassuring and consistent with the low hypercalcemia rate (2.6%) observed in a meta-analysis of data from pediatric vitamin D trials [41]. Similarly, in their pilot RCT of children undergoing surgery for Tetralogy of Fallot, Sahu and colleagues did not report higher blood calcium levels in the arm receiving a 10,000-IU/kg pre-operative loading dose [27]. Second, we observed similar intraoperative and post-operative hypercalciuria rates in the high-dose and usual care arms. While hypercalciuria rates were similar between the groups at 25%, the observed rate was considerably higher than the pooled 2.5% rate reported in the systematic review of pediatric vitamin D clinical trial data [42]. While high in comparison with relatively healthy populations, this difference might be anticipated in the post-operative CHD population due to systematic inflammation, renal dysfunction, and medication use (e.g., diuretics). Comparable findings were presented in the VITdAL-ICU trial (*n* = 475), where 25% hypercalciuria rates were documented pre- and post-study drug administration in critically ill adults receiving either placebo or a 540,000-IU cholecalciferol loading dose [23]. Similar results were evident in the study by Sahu and colleagues where the average perioperative urine calcium to creatine ratios were reported to be similar or potentially lower in the loading dose arm (2.1 vs. 1.0, *p* = 0.16) [27]. In the critical care setting, hypercalciuria may not represent a great biomarker of excess vitamin D given the high prevalence of acute kidney injury and medications (e.g., furosemide) known to promote urinary calcium loss. Further supporting the safety of this regimen was the absence of any definitive cases of nephrocalcinosis or vitamin D-related serious adverse events.

Additional observations related to the ascertainment of the feasibility of a larger phase III RCT were encouraging. First, the high consent rate suggests that patients and families are concerned about post-operative vitamin D deficiency and see value in a trial evaluating whether optimization of vitamin D status enhances recovery after surgery. Second, while our study accrual rate was below the target of 2 to 2.5 patients per month, it still met our feasibility threshold of 1.5 participants per month. Comparison with the literature demonstrates this rate to be consistent with other large trials of hormonal therapy in the pediatric ICU setting [43, 44] and large interventional adult critical care research [45]. Third, we documented an acceptable patient withdrawal rate (11%). While slightly above the acceptable threshold set at 10%, detailed inspection reveals that a multicenter trial that allowed for the transfer of patients between participating centers would eliminate half of the withdrawals observed in this study.

An important observation that would affect the design of a future larger trial was the observation that 43% of the HD arm had post-operative serum 25OHD levels below 50 nmol/L. While data from other pediatric populations suggests the Institute of Medicine high-dose regimen should raise 25OHD concentrations well above our pre-operative target of 90 nmol/L, a more detailed evaluation of these trials demonstrates that concentration achieved is time-dependent, with at least a month (or two) of regular intake necessary to achieve steady state [28, 29]. Despite significant efforts by our research staff to recruit early, only 50% and 25% of study participants were enrolled more than 30 and 60 days prior to surgery, respectively. Consistent with these previous observations, only the subgroup of HD participants receiving more than 30 days of study drug achieved an average post-operative 25OHD concentration above 50 nmol/L (68 nmol/L). Altogether, these observations show that the dosing regimen used in our study can successfully prevent post-operative vitamin D deficiency provided that the duration of supplementation is adequate. Therefore, we recommend that the subsequent trial use modified eligibility criteria to exclude patients whose CHD surgery is scheduled within the following 30 days and therefore cannot receive at least 30 doses pre-operatively. For the subgroup of patients with short intervals between presentation, diagnosis, and surgery, we recommend considering an alternative dosing strategy, such as a loading dose timed with pre-operative anesthesia assessments. Fortunately, multiple adult and pediatric studies have shown that it is possible to safely significantly raise 25OHD levels over a 48- to 72-h time period using a single large age or weight-based enteral load of cholecalciferol (50,000–400,000 IU) [42, 46–48], without any signs of toxicity. In addition to the aforementioned study by Sahu and colleagues, we have confirmed these findings in our recent VITdAL-PICU pilot trial using a loading dose of vitamin D (10,000 IU/kg, maximum 400,000 IU) in critically ill children (NCT02452762) [27].

This study also included an exploratory analysis comparing common post-surgical ICU clinical outcomes between study arms and identified that patients receiving high-dose vitamin D had a significantly shorter duration of mechanical ventilation and a lower rate of acute renal failure. Importantly, the difference in clinical outcomes occurred despite the aforementioned observation that 43% of the high-dose arm did not achieve the target post-operative 25OHD concentration of 50 nmol/L. The suggestion of benefit across multiple outcomes is consistent with vitamin D’s established role as a pleiotropic hormone essential to the proper functioning of multiple organs relevant to the development and recovery of critical illness [6]. In their pilot RCT evaluating pre-operative loading dose vitamin D, Sahu and colleagues demonstrated reductions in the average duration of inotropes and ICU stay, although the study was not powered to evaluate clinical outcomes and statistical significant was not achieved [27]. In addition, there are some potentially relatable pediatric and adult studies that suggest a benefit to high-dose supplementation. For example, a pilot small RCT (*n* = 40) evaluating 2000 IU per day of vitamin D3 in a pediatric cohort with stable congestive heart failure reported statistically significant improvements in clinical symptoms and echocardiography findings [49]. In another pilot trial in the setting of severe pediatric burn, the authors suggested potential benefits for sepsis, bone mass density, keloid, and fracture outcomes with receipt of high-dose vitamin supplementation. More recently, individual adult ICU trials have also suggested increased chance of survival, reduced length of ICU stay, and improvement in long-term functional outcomes [22, 23]. Following the completion of these pilot studies, three systematic reviews and meta-analysis concluded potential benefit [50–52], leading to the initiation of two large ongoing adult multicenter trials focused on this question [24, 25]. The VIOLET study was recently published and did not identify a mortality reduction following a 540,000-IU enteral load in vitamin D-deficient adult patients with acute lung injury, at significant risk for ICU admission [24].

This study had several limitations. First, the study did not achieve the target sample size, due to a combination of limited funding and smaller than expected number of patients referred for surgery during the study window. Second, the duration of the pre-operative study drug administration was shorter than desired. Despite these limitations, the study design was robust enough to demonstrate a statistically significant reduction in post-operative vitamin D deficiency. An important third limitation is that the trial was not adequately powered to appropriately evaluate for the differences in clinical outcomes, and the results of our exploratory analysis should be considered hypothesis-generating. Given the small numbers involved, any differences (or lack thereof) between the groups could represent type I or type II errors. Finally, this study was conducted in a single Canadian tertiary center that does not perform surgery on pediatric patients with more complex lesions (e.g., risk adjustment in congenital heart surgery 5 or 6) [53]. As a result, findings from this study may not apply to other institutions performing more complex CHD surgeries.

## Conclusions

Recent evidence suggests that many children are vitamin D deficient following cardiac surgery, and this could contribute to greater illness severity. Phase III clinical trials evaluating whether optimization of post-operative vitamin D status improves clinical outcomes have been suggested. However, there was considerable uncertainty about the feasibility of such a trial, including whether the high-dose supplementation regimen recommended by the Institute of Medicine would safely prevent vitamin D deficiency in this high-risk population. In this study, we determined that the high-dose regimen was able to significantly reduce the prevalence of vitamin D deficiency without evidence of additional adverse events or toxicity. While the vitamin D results largely support the feasibility of the dosing protocol, observations that the children who received study drug for less than 30 days do not sufficiently elevate vitamin D concentrations  suggest these patients should either be excluded or receive an additional loading dose at the time of randomization. Vitamin D findings, combined with observations of both high consent rates and low rates of study withdrawal and protocol non-adherence, further support the feasibility of a large multicenter phase III trial focused on the clinical outcome.

## Supplementary information

**Additional file 1.** CONSORT checklist. CONSORT 2010 checklist of information to include when reporting a pilot or feasibility trial.

**Additional file 2.** Timing of research sample collection. Detailed schema of the timing of research sample collection in relation to other study and clinical procedures.

**Additional file 3.** Age-specific thresholds for elevated calcium-creatinine ratio. Age-specific thresholds for elevated calcium:creatinine ratio used to define hypercalciuria in this trial.

**Additional file 4.** Summary of Protocol Deviations. Detailed listing of protocol deviations that occurred during the conduct of this trial.

## Data Availability

The datasets used and/or analyzed during the current study are available from the corresponding author on reasonable request.

## Abbreviations

25OHD: 25-Hydroxyvitamin D
CONSORT: Consolidated Standards of Reporting Trials
CHD: Congenital heart disease
CI: Confidence interval
HD: High dose
ICU: Intensive care unit
IQR: Interquartile range
POD: Post-operative day
PRISM: Pediatric Risk of Mortality
RCT: Randomized controlled trial
UC: Usual care
VITdAL-ICU: Correction of Vitamin D Deficiency in Critically Ill Patients
VITdAL-PICU: Rapid Normalization of Vitamin D in Critically Ill Children: a Phase II Dose Evaluation Randomized Controlled Trial
