# Supplementary figures and images for: Prevention of post-cardiac surgery vitamin D deficiency in children with congenital heart disease: a pilot feasibility dose evaluation randomized controlled trial
Source: Pilot Feasibility Stud. 2020 Oct 22;6:159. doi: 10.1186/s40814-020-00700-3 (PMC7583219; doi:10.1186/s40814-020-00700-3)

Additional File 2: Timing of Research Sample Collection

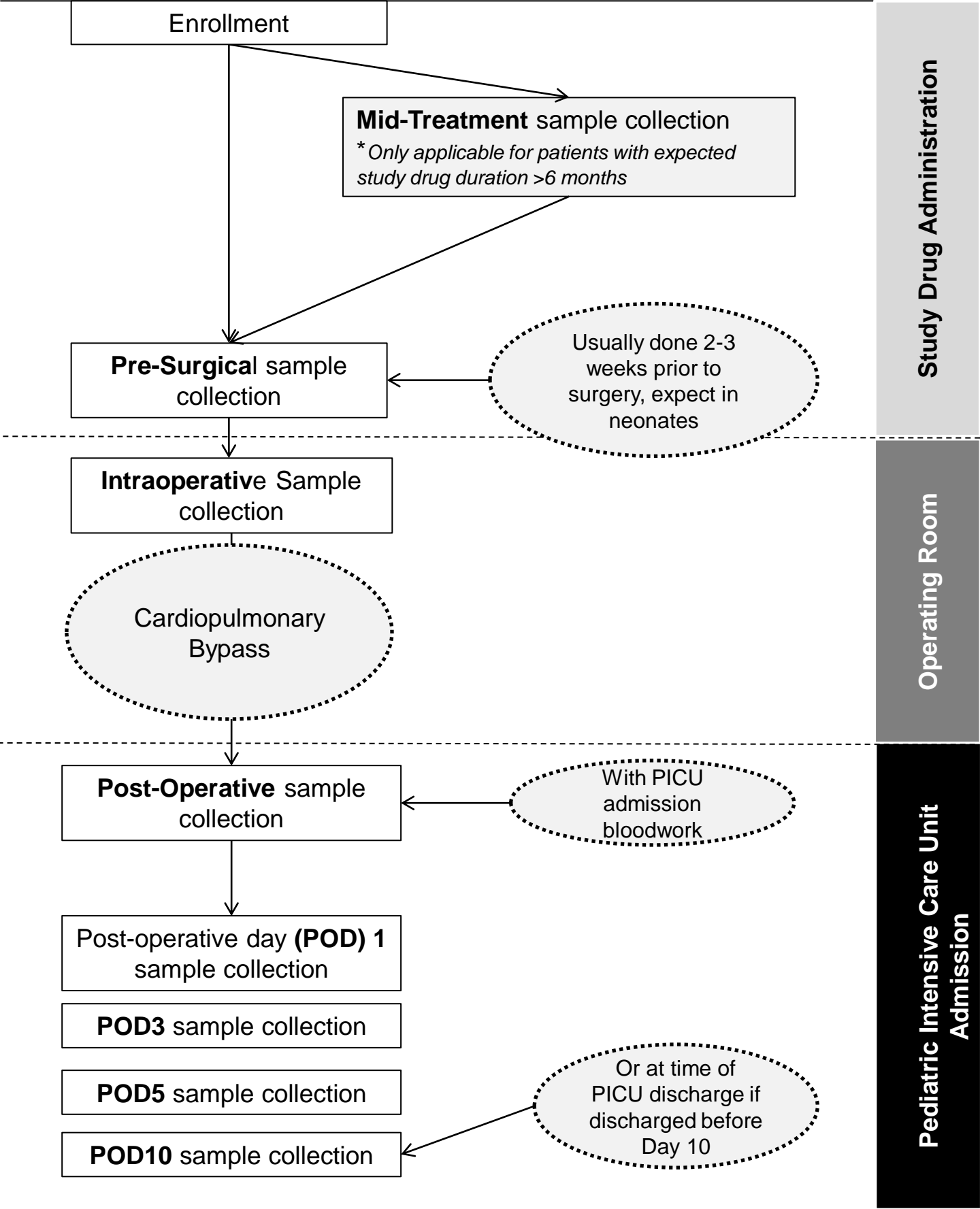

Supplement: Supplementary file 2 — Additional file 2. Timing of research sample collection. Detailed schema of the timing of research sample collection in relation to other study and clinical procedures. [file 40814_2020_700_MOESM2_ESM.pdf]
