# Supplementary material for: Prevention of post-cardiac surgery vitamin D deficiency in children with congenital heart disease: a pilot feasibility dose evaluation randomized controlled trial
Source: Pilot Feasibility Stud. 2020 Oct 22;6:159. doi: 10.1186/s40814-020-00700-3 (PMC7583219; doi:10.1186/s40814-020-00700-3)
Supplement: Supplementary file 3 — Additional file 3. Age-specific thresholds for elevated calcium-creatinine ratio. Age-specific thresholds for elevated calcium:creatinine ratio used to define hypercalciuria in this trial. [file 40814_2020_700_MOESM3_ESM.docx]

**Additional File 3 -Age specific thresholds for elevated calcium-creatinine ratio**

| **Age (year)** | **95^th^ % Ca/Cr ratio (mmol/mmol)** |
| --- | --- |
| **< 1** | **2.2** |
| **1-2** | **1.5** |
| **2-3** | **1.4** |
| **3-5** | **1.1** |
| **5-7** | **0.8** |
| **7-17** | **0.7** |
