# Supplementary material for: Prevention of post-cardiac surgery vitamin D deficiency in children with congenital heart disease: a pilot feasibility dose evaluation randomized controlled trial
Source: Pilot Feasibility Stud. 2020 Oct 22;6:159. doi: 10.1186/s40814-020-00700-3 (PMC7583219; doi:10.1186/s40814-020-00700-3)
Supplement: Supplementary file 4 — Additional file 4. Summary of Protocol Deviations. Detailed listing of protocol deviations that occurred during the conduct of this trial. [file 40814_2020_700_MOESM4_ESM.docx]

**Additional File 4: Summary of Protocol Deviations**

All protocol deviations were reviewed by the Principal Investigator and where relevant, the study safety officer and/or nephrologist. No patients were withdrawn because of a protocol deviation, and all deviations were determined to have no impact on patient safety, patient rights, or integrity of study data. All deviations were reported to the CHEO Research Ethics Board.

| **Participant** | **Type of Protocol Deviation** | **Description of Protocol Deviation** |
| --- | --- | --- |
| SP04 | Study procedures conducted out of sequence | Pre-surgical research bloodwork for analysis of 25OHD not ordered at time of pre-surgical appointment. Study coordinator was able to obtain discard blood to store for later analysis. Pre-surgical appointment was the day before surgery, so the 25OHD result would not have been reviewed by the safety officer pre-operatively even if it had been ordered. |
| SP06 | Study procedures omitted | Upon review of DSMB table, study staff became aware that SP06 did not have an ultrasound despite an elevated intraoperative calcium:creatinine ratio. It was determined that this event posed no risk to patient safety through review of the number of study drug doses taken (<3), the POD 1 urine calcium:creatinine ratio (which was normal), and consultation with nephrology. |
| SP12 | Study procedures omitted | POD 1 urine sample could not be collected (low urine output) |
| SP16 | Study procedures omitted | Intraoperative urine sample could not be collected. Requisition and sample container given to OR nurses as per usual protocol, no sample obtained. Unclear at this time why sample was not collected. POD 1 urine sample was collected and reviewed. POD1 urine calcium:creatinine ratio was below the threshold for hypercalciuria. |
| SP46 | Study procedures omitted | POD 1 urine could not be collected as patient had low urine output. Study coordinator attempted to collect a urine sample the following day but unable to obtain sample. Intraoperative urine level was normal and patient only received ~3 weeks of study drug. Nephrology consulted. No safety concerns. |
| SP28 | Study procedures omitted | POD 1 urine calcium:creatinine ratio was above the threshold for hypercalciuria. Nephrology asked for a repeat urine calcium:creatinine but patient was discharged before it could be obtained. The study team attempted to get the repeat urine at a post-operative appointment, but the patient was not able to give a sample. |
| SP06 | Study drug and/or calendar not returned | Study drug not returned. Family asked and reminded to bring bottle next time they came in to CHEO, but it was not returned. |
| SP12 | Study drug and/or calendar not returned | Study drug not returned. Family asked and reminded to bring bottle next time they came in to CHEO, but it was not returned. |
| SP14 | Study drug and/or calendar not returned | Mother did not bring unused drug to surgical appointment and was asked to return it next time she was at CHEO. At subsequent visit, mother returned one unopened bottle to CHEO, but was not able to find the partial unused bottle. The study calendar was also not returned. |
| SP46 | Study drug and/or calendar not returned | Study drug not returned. Family asked and reminded to bring bottle next time they came in to CHEO, but it was not returned. |
| SP01 | Study drug and/or calendar not returned | Family did not return study calendar. Reminded to bring it next time they were at CHEO but was not returned. |
| SP04 | Study drug and/or calendar not returned | Family did not return study calendar. Reminded to bring it next time they were at CHEO but was not returned. |
| SP27 | Study drug and/or calendar not returned | Family did not return study calendar. Reminded to bring it next time they were at CHEO but was not returned. |
| SP35 | Study drug and/or calendar not returned | Family did not return study calendar. Reminded to bring it next time they were at CHEO but was not returned. |
| SP37 | Study drug and/or calendar not returned | Family did not return study calendar. Reminded to bring it next time they were at CHEO but was not returned. |
| SP46 | Study drug and/or calendar not returned | Family did not return study calendar. Reminded to bring it next time they were at CHEO but was not returned. |
| SP19 | Medication dispensing error | SP19 had surgery on 09-Jun-2014. On the morning of surgery, the Cardiovascular Surgery nurse took the unused study drug from the bedside and gave it to the study coordinator. The study coordinator returned the unused drug to pharmacy. On 18-Jun-2014, study staff was notified that SP19 had received study drug post-operatively on 9-11 June and 13-15 June. The Principal Investigator reviewed the patient chart and discovered that a resident had written an order to administer vitamin D as per study and a new bottle was dispensed. The additional doses were determined to pose no risk to the patient's safety and would not affect the integrity of the data. The patient had only received 3 doses of study drug prior to surgery and had no adverse events attributable to vitamin D pre- or post-operatively. |
| SP26 | Medication dispensing error | Patient was enrolled into the study as an inpatient and was administered a dose of standard issue vitamin D given to all inpatients instead of the study drug. |
| SP12 | Other | The family did not return drug and calendar at time of surgery. The family was reminded to return calendar and drug at the next appointment. The family returned the calendar at next appointment, but not the study drug. The calendar was reviewed, and it was noted that the patient received 5 doses of the study drug post-operatively on Feb. 23 to 27. The family was contacted to ensure they were no longer administering the study drug. Given the short duration on study drug pre-operatively, it was determined that this was not a risk to the patient's safety. |
| SP14 | Other | The Principal Investigator entered two SAE Reports into REDCap on 24-Jun-2013. At the time the report was entered, the report was not e-signed. Study staff became aware of this on 20-Jul-2014. The reports were e-signed on 31-Jul-2014. Given the delay between the time of the SAE event and the time the reports were signed by the Principal Investigator, a protocol deviation report was filed. |
| SP24 | Other | The patient's 25OHD level at her pre-surgical appointment met the study threshold above which study drug is reduced by 50% (Note: this threshold is established only because vitamin D levels above this threshold do not bring any more benefit, not because it is a risk to the patient; blood calcium was well within the normal range). However, the patient's mother reported she was also giving patient an immune tonic from a naturopath which contained an unknown amount of vitamin D. Since the patient would be returning to clinic within a week, it was decided to stop the study drug and re-test the vitamin D level at the next appointment, find out more about the immune tonic, and then determine whether drug could be continued at 50%. The patient was sick on the day of the appointment, so blood could not be collected. The Principal Investigator spoke to mom and found out more about the immune tonic. Mom also expressed she was very concerned the study drug was stopped because she was worried about vitamin D deficiency. The Principal Investigator asked to unblind the patient to determine if the increase in vitamin D level was because patient was in the high-dose group (in which case drug would be continued at 50% as per the study protocol) or if the increased vitamin D level could potentially be related to the immune tonic (if patient was in the low dose group). The patient was in the high-dose group so study drug was continued at a reduced dose (50%). |

25OHD – 25-hydroxyvitamin D; DSMB – Data Safety Monitoring Board; POD – post-operative day; CHEO – Children’s Hospital of Eastern Ontario; SAE – Serious Adverse Event
